# Increased Prevalence of Elevated D-Dimer Levels in Patients on Direct Oral Anticoagulants: Results of a Large Retrospective Study

**DOI:** 10.3389/fcvm.2022.830010

**Published:** 2022-03-31

**Authors:** Sara Reda, Elena Thiele Serra, Jens Müller, Nasim Shahidi Hamedani, Johannes Oldenburg, Bernd Pötzsch, Heiko Rühl

**Affiliations:** Institute of Experimental Hematology and Transfusion Medicine, University Hospital Bonn, Bonn, Germany

**Keywords:** D-dimer, direct oral anticoagulants, venous thromboembolism, vitamin K antagonists, secondary prophylaxis

## Abstract

Elevated D-dimer levels during anticoagulant therapy with vitamin K antagonists (VKA) are associated with an increased risk of thrombosis. It has been hypothesized that elevated D-dimer levels in patients receiving direct oral anticoagulants (DOACs) also indicate an increased risk of thrombosis recurrence, but data on the distribution of D-dimer levels in patients with VTE on DOACs are sparse. In the present study we retrospectively analyzed D-dimer levels in patients taking DOACs after first or recurrent venous thrombosis (*n* = 1,716, 1,126 thereof rivaroxaban, 481 apixaban, 62 edoxaban, and 47 dabigatran). Patients on VKA (*n* = 402) served as control group. Thrombotic events in the study population were categorized into distal deep venous thrombosis (DVT, *n* = 552 patients), distal DVT with pulmonary embolism (PE, *n* = 166), proximal DVT (*n* = 685), proximal DVT with PE (*n* = 462), PE without DVT (*n* = 522), DVT of the upper extremity (*n* = 78), cerebral venous sinus thrombosis (CVST, *n* = 48), and other venous thrombosis (*n* = 74). In VKA users a median D-dimer level of 0.20 mg/l was observed. In patients on DOACs D-dimer levels were significantly higher, with 0.26 mg/l for rivaroxaban, 0.31 mg/l for apixaban (*P* < 10^−16^ each), 0.24 mg/l for edoxaban (*P* = 2 × 10^−5^), and 0.25 mg/l for dabigatran (*P* = 4 × 10^−4^). These differences in comparison to patients on VKA treatment could not be explained by the patients' age, sex, body mass index, and type of thrombosis as these characteristics did not differ significantly between cohorts. Moreover, the prevalence of D-dimer levels above age-adjusted cut-offs [≥0.50 mg/l in ≤50-year-old patients, ≥(age × 0.01) mg/l in >50-year-old patients] was higher in patients on rivaroxaban (13.9%, RR 1.74, 95% CI 1.21–2.50), apixaban (17.0%, RR 2.14, 95% CI 1.45–3.15) and dabigatran (23.4%, RR 2.94, 95% CI 1.59–5.44) than in patients on VKA (8.0%). In patients on edoxaban D-dimer levels above the reference range were observed in 14.5%, but no statistical significance was reached in comparison to the VKA cohort. In conclusion, the obtained data suggest, that the type of oral anticoagulant should be considered in the clinical assessment of D-dimer levels in thrombosis patients. Further studies are warranted to evaluate a potential association between elevated D-dimer levels and thrombosis risk in patients on DOACs.

## Introduction

D-dimer molecules are generated when cross-linked fibrin is digested by plasmin during fibrinolysis (1). A prerequisite of D-dimer generation is the formation of thrombin upon activation of the clotting cascade. Thrombin converts fibrinogen to fibrin monomers and activates factor XIII which cross-links the D domains of adjacent fibrin monomers resulting in cross-linked fibrin (2). Therefore, D-dimer is a compound biomarker of activation of coagulation and fibrinolysis, which can be measured using monoclonal antibodies specific for an epitope on cross-linked D-dimer molecules that is not present on fibrinogen or fibrin monomers (2, 3).

Measurements of D-dimer are widely applied in diagnostic algorithms for exclusion of acute venous thromboembolism (VTE) including deep venous thrombosis (DVT) (4, 5) and pulmonary embolism (PE) (6), but also in the diagnosis of other acute hypercoagulable states such as disseminated intravascular coagulation (7, 8). Furthermore, elevated D-dimer levels are used as predictive marker for an increased risk of VTE recurrence in patients under anticoagulant therapy with vitamin K antagonists (VKA) (9–12). A number of decision algorithms for continuation or withdrawal of VKA have been proposed, that include D-dimer testing during VKA treatment (9–16), often combined with additional measurements after discontinuation of VKA (10, 11, 15, 16). With increasing use of direct oral anticoagulants (DOACs) replacing VKA in the treatment of VTE, the question arises whether these decision algorithms can be also applied to patients under DOAC therapy. A prerequisite of this would be knowledge on the distribution of D-dimer levels in patients with VTE on DOACs, but these data are sparse. Results from one previous retrospective case-control study suggested, that patients with VTE under treatment with DOACs have a higher rate of elevated D-dimer levels in comparison to patients on VKA (17). However, higher rates of elevated D-dimer levels were also observed after withdrawal of anticoagulant treatment, and absolute values of D-dimer measurements were not reported (17).

## Materials and Methods

### Identification and Inclusion of Patients

This retrospective cohort study was conducted at the Institute of Experimental Hematology and Transfusion Medicine, Bonn, Germany in thrombosis patients who were referred to the outpatient clinic for coagulation disorders of our institution between 2008 and 2019. The patients were referred in the context of thrombophilia testing in the absence of suspected or confirmed acute thrombosis. Demographic and clinical data were extracted from the medical records of patients who received oral anticoagulant therapy. Data on D-dimer testing were retrieved from the database of the laboratory information system.

**Figure 1:**
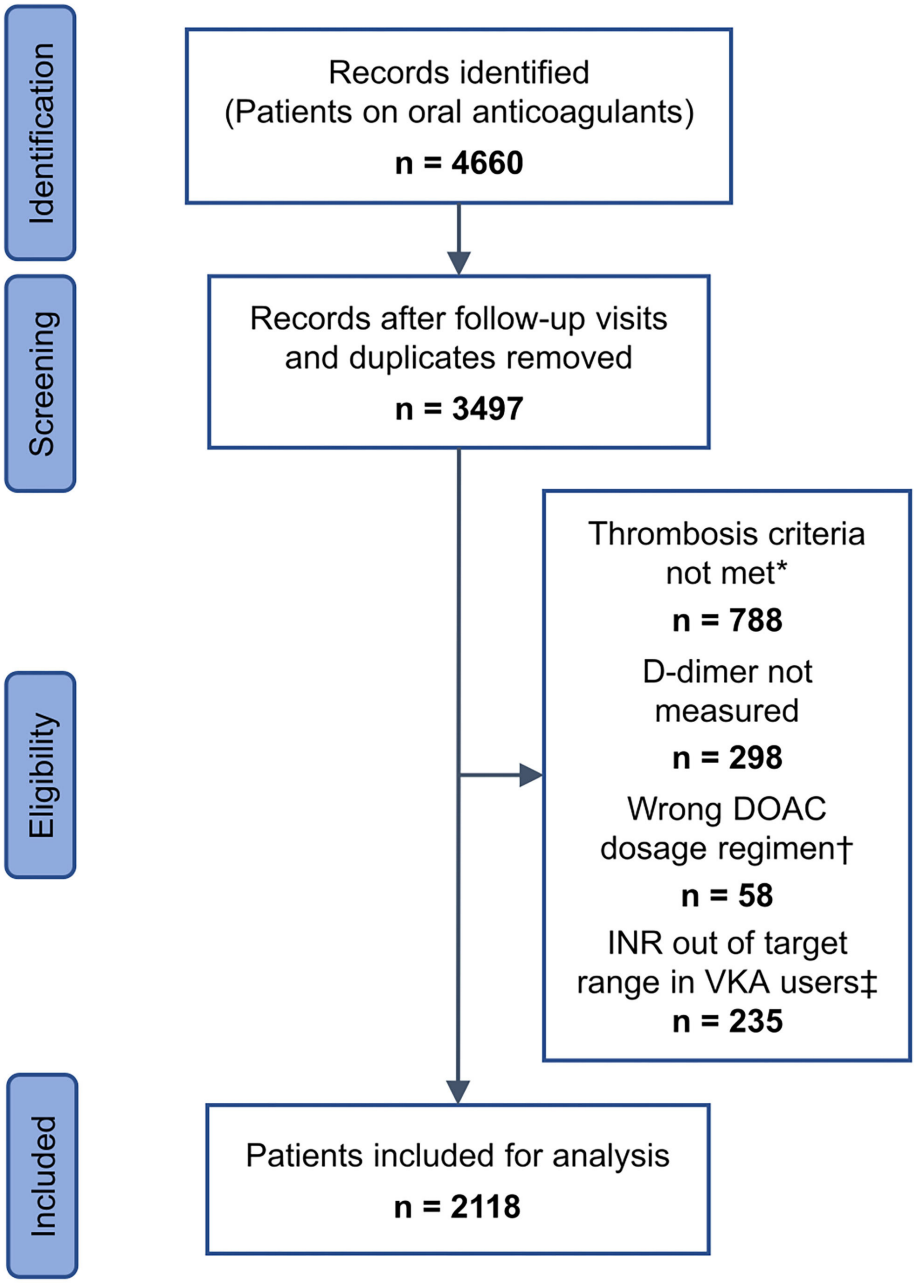
Identification and inclusion of patient data. *Arterial thrombosis, retinal vein thrombosis, superficial venous thrombosis, or any thrombosis within one month preceding blood sampling. †Any dosage regimen other than apixaban 5 or 2.5 mg b.i.d. (*bis in die*, twice daily), dabigatran 150 or 110 mg b.i.d., edoxaban 60 or 30 mg q.d. (*quoque die*, once daily), and rivaroxaban 20 or 10 mg q.d., ‡ < 2.0 (*n* = 142) or > 3.0 (n = 93). DOAC, direct oral anticoagulant; INR, international normalized ratio; VKA, vitamin K antagonist.

In the above-mentioned 12-year period, a total number of 4,660 referrals of patients on oral anticoagulants were identified, thereof 3,497 first-time referrals, whose records were further screened. Patients were included, if they received either VKA, apixaban 5 or 2.5 mg b.i.d. (*bis in die*, twice daily), dabigatran 150 or 110 mg b.i.d., edoxaban 60 or 30 mg q.d. (*quoque die*, once daily), or rivaroxaban 20 or 10 mg q.d., if they had a history of venous thrombosis, and if D-dimer testing was performed at the visit. Exclusion criteria were single or additional anticoagulant therapy with any drug or dosage other than listed above, any thrombotic event within one month preceding blood sampling, and arterial thrombosis, retinal vein thrombosis, or superficial venous thrombosis as the sole thrombotic event in a patient. Additional exclusion criteria for patients receiving VKA were an international normalized ratio (INR) outside the therapeutic target range of 2.0–3.0. Of the 3,497 first-time referrals, 788 patients were excluded from the study population because they did not meet the thrombosis criteria, 298 patients because D-dimer testing was not performed, and 58 patients because of an anticoagulant dosage regimen not fulfilling the requirements. In 142 patients on VKA the INR was < 2.0, and in 93 patients the INR was > 3.0. The remaining 2,118 patients were included in the final analysis (Figure 1).

### D-Dimer and INR Testing

Blood samples were obtained from a venipuncture of a suitable arm vein using a 21-gauge winged infusion set (Sarstedt, Nümbrecht, Germany). After discarding the first 2 ml, blood was drawn into citrate tubes (10.5 mmol/l final concentration, Sarstedt). Plasma samples were obtained by centrifugation (2,600 x g, 10 min) within one hour after blood draw and assayed within 4 h. D-dimer testing was performed using the Innovance^TM^ D-dimer assay on the BCS^®^ XP analyzer, the INR was assayed using the Innovin reagent (Dade Behring—A Siemens Company, Marburg, Germany). Innovance D-dimer is a microparticle-enhanced immunoassay using a monoclonal antibody (8D3, Dade Behring) for D-dimer binding (18). Preanalytics, INR and D-dimer measurement in the laboratory of our institution were covered by accreditation with the national accreditation body and were performed according to ISO standards.

### Statistical Analysis

Data are generally presented as median and interquartile range (IQR). Normality of data was tested using the Shapiro-Wilk test. A Kruskal-Wallis test was performed, followed by a two-tailed Mann-Whitney test, to compare the patients' age, BMI, and D-dimer levels obtained in the different cohorts of the study population. *P* values ≤ 0.05 were considered significant. The Bonferroni method was applied to correct for multiple testing and was performed for four comparisons to compare each of the four cohorts using DOACs (rivaroxaban, apixaban, edoxaban, dabigatran) to the VKA cohort, and for six comparisons to additionally compare the subgroups using rivaroxaban and apixaban at different dosages to the VKA cohort. The chi-square test was used for the comparison of proportions. When comparing the proportions of patients with different thrombotic events in the five cohorts using VKA or DOACs. Monte Carlo simulation was used, followed by the Marascuilo procedure, to identify the proportions possibly responsible for rejecting H_0_; i.e., the cohorts in which the proportion of patients with a specific thrombotic event differed significantly from those in the other cohorts. All calculations were performed using the XLSTAT statistical and data analysis solution software (Addinsoft, Boston, MA, USA). All authors had access to primary data.

## Results

### Study Population and Anticoagulant Medication

Included over a 12-year period, the final study population consisted of 2,118 patients (1,005 males, 1,113 females) with a history of VTE or other venous thrombosis, thereof 481 patients on apixaban (270 taking 5 mg b.i.d., 211 taking 2.5 mg b.i.d.), 47 on dabigatran (33 taking 150 mg b.i.d., 14 taking 110 mg b.i.d.), 62 on edoxaban (54 taking 60 mg q.d., 8 taking 30 mg q.d.), and 1,126 patients on rivaroxaban (935 taking 20 mg q.d., 191 taking 10 mg q.d.). The control group of VKA users consisted of 402 patients (Table 1). Overall, the patients' median age was 54 (IQR 41–65) years.

**Table 1:** Patient characteristics and D-dimer levels in the study population.

**Drug**	**Patients, n**	**Sex (m/f), n**	**Age, years**	**BMI, kg/m^**2**^**	**D-dimer, mg/l**	P^*^
VKA	402	203/199	53 (39–65)	28 (25–31)	0.20 (0.17–0.29)	-
Rivaroxaban (total)	1,126	557/569	54 (41–65)	27 (24–31)	0.26 (0.19–0.40)	<10^−16^
20 mg q.d.	935	475/460	53 (40–65)	27 (24–31)	0.26 (0.19–0.40)	6 × 10^−4^
10 mg q.d.	191	82/109	55 (42–66)	27 (24–31)	0.28 (0.19–0.46)	2 × 10^−14^
Apixaban (total)	481	194/287^†^	55 (42–67)	27 (24–31)	0.31 (0.20–0.48)	<10^−16^
5 mg b.i.d.	270	113/157	53 (40–65)	27 (24–32)	0.30 (0.19–0.47)	<10^−16^
2.5 mg b.i.d.	211	81/130	57 (43–69)	26 (24–30)	0.33 (0.22–0.49)	<10^−16^
Edoxaban	62	32/30	53 (43–65)	27 (24–30)	0.24 (0.19–0.47)	2 × 10^−5^
Dabigatran	47	19/28	57 (45–71)	27 (24–30)	0.25 (0.19–0.64)	4 × 10^−4^

*Age, BMI, and D-dimer are presented as median and interquartile range*.

* *D-dimer levels in comparison to the VKA cohort were calculated using the Mann-Whitney test, P values < 0.05 were considered significant*.

† *The male/female ratio was compared to the VKA cohort by chi-square analysis with P = 0.010 for apixaban (total) vs. VKA, and P ≥ 0.05 for all other comparisons. b.i.d., bis in die (twice daily); BMI, body mass index; f, female; m, male; NS, not significant; q.d., quoque die (once daily); VKA, vitamin K antagonist*.

### D-Dimer Levels Are Higher in Patients on DOACs Than in VKA Users

**Figure 2:**
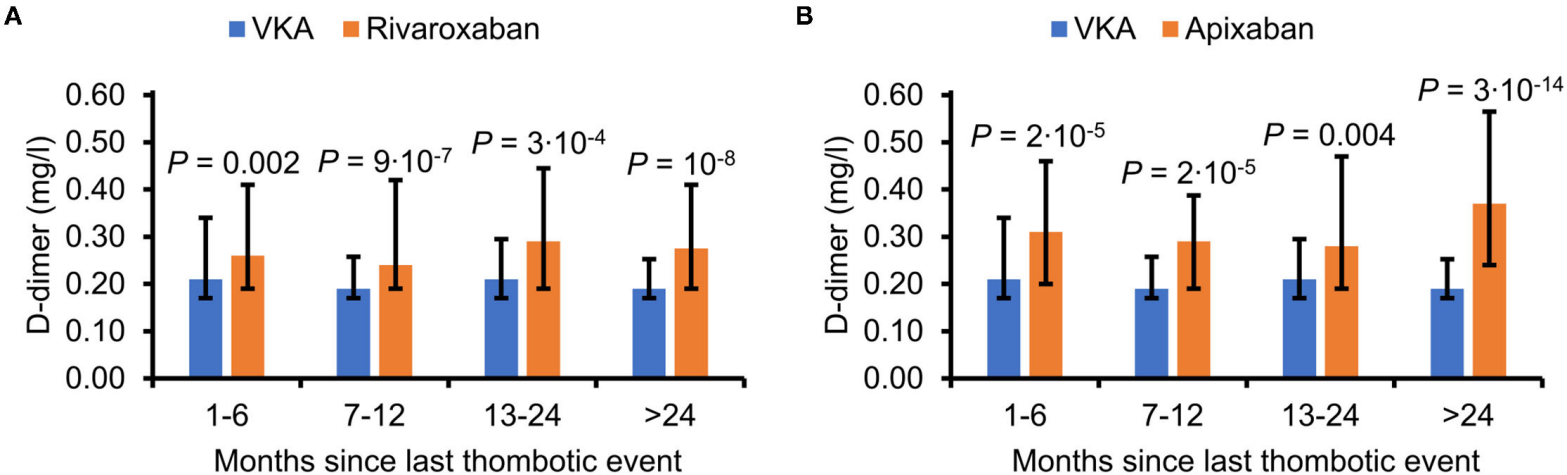
D-dimer levels according to the time since the last venous thrombotic event. The study population was categorized into patients in whom D-dimer levels were measured 1–6 months (137 patients on VKA, 573 on rivaroxaban, 183 on apixaban), 7–12 months (86 VKA, 233 rivaroxaban, 82 apixaban), 13–24 months (67 VKA, 147 rivaroxaban, 87 apixaban), and > 24 months (112 VKA, 172 rivaroxaban, 130 apixaban) since the last event. Data are shown as median and interquartile range. *P* values indicate differences between patients on VKA and **(A)** rivaroxaban or **(B)** apixaban and were calculated using the Mann-Whitney test. VKA, vitamin K antagonist.

In VKA users D-dimer levels of 0.20 (0.17–0.29) mg/l and an INR of 2.2 (2.4–2.7) were observed. In patients on DOACs D-dimer levels were significantly higher, with 0.26 (0.19–0.40) mg/l for rivaroxaban, 0.31 (0.20–0.48) mg/l for apixaban, 0.24 (0.19–0.47) mg/l for edoxaban, and 0.25 (0.19–0.64) mg/l for dabigatran. In a more detailed comparison including the subgroups of patients taking different dosages of rivaroxaban or apixaban with the control group of patients on VKA, D-dimer levels in comparison to VKA were also higher (Table 1). In order to analyze if the interval since the last thrombotic event affected the observed differences in D-dimer levels between patients on rivaroxaban or apixaban and patients taking VKA, these cohorts were further categorized into patients with an interval of 1–6, 7–12, 13–24 months, and more than 24 months since the last thrombotic event. As shown in Figure 2, D-dimer levels were constantly higher in patients on apixaban or rivaroxaban in comparison to patients on VKA throughout all four categories of time intervals.

### Increased D-Dimer Levels in DOAC Users Are Not Explained by Age, Sex, BMI, or Type of Thrombosis

In order to analyze if the observed differences in D-dimer levels between DOAC and VKA users could be explained by differences in age, sex, BMI, or different frequencies of types of thrombosis, the cohorts of patients using different anticoagulant drugs were analyzed for differences regarding these features. Overall, the patients' age in the four cohorts of patients on apixaban, dabigatran, edoxaban, and rivaroxaban did not differ to the patients' age in the VKA cohort. The male/female ratio in the DOAC cohorts and dosage-specific subgroups did also not differ significantly to the VKA cohort. Thrombotic events in the study population were categorized into distal DVT (prevalent in 552 patients), distal DVT with PE (*n* = 166), proximal DVT (*n* = 685), proximal DVT with PE (*n* = 462), PE without DVT (*n* = 522), DVT of the upper extremity (*n* = 78), cerebral venous sinus thrombosis (CVST, *n* = 48), and other venous thrombosis (*n* = 74). Overall, 666 patients had a history of recurrent VTE. Table 2 shows the frequencies of patients with these types of thrombosis in the cohorts of DOAC and VKA users. Chi-square analysis revealed a greater proportion of patients with PE without DVT in the apixaban cohort (142/481, 30%) than in the VKA cohort (76/402, 19%, *P* = 0.003), but the proportions of patients with any PE did not differ between cohorts. Additionally, the proportion of patients with CVST in the edoxaban cohort (0/62) was significantly lower than in the VKA cohort (18/402, 4%, *P* = 4 × 10^−6^). No other statistically significant differences between the proportions of the different types of thrombosis in the different cohorts were observed.

**Table 2:** Thrombotic events in the study population.

**Patients with thrombotic events in cohort**	VKA, n (%)^*^	**Rivaroxaban, n (%)**	**Apixaban, n (%)**	**Edoxaban, n (%)**	**Dabigatran, n (%)**	**Total, n (%)**	***P* ^†^**
Distal DVT	113 (28%)	280 (25%)	124 (26%)	22 (35%)	13 (28%)	552 (26%)	0.325
Proximal DVT	119 (30%)	357 (32%)	162 (34%)	28 (45%)	19 (40%)	685 (32%)	0.090
DVT of upper extremity	16 (4%)	44 (4%)	16 (3%)	2 (3%)	0	78 (4%)	0.687
Proximal DVT with PE	102 (25%)	240 (21%)	98 (20%)	9 (15%)	13 (28%)	462 (22%)	0.158
Distal DVT with PE	33 (8%)	100 (9%)	28 (6%)	4 (6%)	1 (2%)	166 (8%)	0.149
PE without DVT	76 (19%)	275 (24%)	142 (30%)	20 (32%)	9 (19%)	522 (25%)	**0.003**
PE (any of the above)	201 (50%)	591 (52%)	255 (53%)	31 (50%)	21 (45%)	1,099 (52%)	0.727
CVST	18 (4%)	14 (1%)	11 (2%)	0	5 (11%)	48 (2%)	4 × 10^−6^
Other venous thrombosis	16 (4%)	34 (3%)	17 (4%)	2 (3%)	5 (11%)	74 (3%)	0.086
Recurrent VTE	132 (33%)	331 (29%)	158 (33%)	28 (45%)	17 (36%)	666 (31%)	0.256

* *n indicates the absolute number of patients with a history of the listed thrombotic events, % indicates the respective proportions in the cohorts on different anticoagulant therapies*.

† *P describes differences between the proportions of patients with listed thrombotic events in the cohorts. In the case of significant differences, (P < 0.05) the responsible proportion is shown in bold. CVST, cerebral venous sinus thrombosis; DVT, deep venous thrombosis; PE, pulmonary embolism; VKA, vitamin K antagonist; VTE, venous thromboembolism*.

### D-Dimer Levels Above the Cut-Off Are More Frequent in DOAC Users Than in VKA Users

In addition to the comparison of the absolute values of D-dimer levels in the patients' plasma, we compared the frequencies of D-dimer levels above age-adjusted cut-off values in the cohorts and dosage-specific subgroups of the study population. For this analysis, D-dimer levels ≥ 0.50 mg/l in ≤ 50-year-old patients, and ≥ (age × 0.01 mg/l) in > 50-year-old patients were defined as cut-offs, which had been validated for exclusion of deep vein thrombosis in a previous study (19). As is shown in Table 3, the prevalence of D-dimer levels above these cut-offs was higher in patients on rivaroxaban, apixaban and dabigatran than in VKA users, with frequencies of 13.9, 17.0 and 23.4%, respectively, in comparison to 8.0%. Furthermore, the risk of a D-dimer level above the cut-off was significantly increased in the rivaroxaban cohort (risk ratio 1.74, 95% confidence interval (CI) 1.21–2.50), apixaban cohort (risk ratio 2.14, 95% CI 1.45–3.15) and dabigatran cohort (risk ratio 2.94, 95% CI 1.59–5.44) in comparison to the VKA cohort. In patients on edoxaban D-dimer levels above the reference range were observed in 14.5%, but no statistical significance was reached in comparison to the VKA cohort. Analysis of the dosage-specific subgroups of DOAC users also revealed a higher prevalence of D-dimer levels above the cut-offs in patients taking rivaroxaban 20 mg q.d., rivaroxaban 10 mg q.d., apixaban 5 mg b.i.d., and apixaban 2.5 mg b.i.d (Table 3). Different dosages of dabigatran and edoxaban were not included in this analysis due to the small sample size in the respective subgroups.

**Table 3:** Prevalence of elevated D-dimer levels according to anticoagulant therapy.

**Drug**	Patients with elevated^*^D-dimer, n	**Thereof ≤ /> 50 years, n**	**Overall frequency in cohort**	**Risk ratio^†^**	**95% confidence interval**
VKA	32	11/21	8.0%	-	-
**Rivaroxaban (total)**	156	57/99	13.9%	1.74	1.21–2.50
20 mg q.d.	121	43/78	12.9%	1.63	1.12–2.36
10 mg q.d.	35	14/21	18.3%	2.30	1.47–3.60
**Apixaban (total)**	82	26/56	17.0%	2.14	1.45–3.15
5 mg b.i.d.	53	17/36	19.6%	2.47	1.64–3.72
2.5 mg b.i.d.	29	9/20	13.7%	1.73	1.07–2.77
Edoxaban	9	2/7	14.5%	1.82	0.92–3.63
Dabigatran	11	1/10	23.4%	2.94	1.59–5.44

* *D-dimer levels ≥ 0.50 mg/l in ≤ 50-year-old patients, and ≥ (age × 0.01) mg/l in > 50-year-old patients*.

† *In comparison to the VKA cohort. b.i.d., bis in die (twice daily); q.d., quoque die (once daily); VKA, vitamin K antagonist*.

## Discussion

The aim of this study was to comparatively assess D-dimer levels in patients with venous thrombosis receiving anticoagulant treatment with VKA and DOACs. Generally, all DOACs currently available on the market in the European Union were associated with higher plasma levels of D-dimer compared with VKA. Moreover, D-dimer levels above an age-adjusted cut-off were found more frequently in patients treated with rivaroxaban, apixaban, and dabigatran than in patients on VKA, thereby strengthening the results of Legnani et al., who observed increased rates of elevated D-dimer in patients with thrombosis during DOAC treatment in comparison to patients treated with VKA (17).

The present study is the first in which absolute values of D-dimer in patients with venous thrombosis on VKA and DOACs were compared. In patients with atrial fibrillation (AF) the effect of DOACs and VKA on D-dimer levels was studied previously with inconsistent results. As in our study in patients with venous thrombosis, D-Dimer levels in patients with AF on apixaban were increased in comparison to VKA users (20, 21). In contrast to our findings, dabigatran in AF was associated with lower D-dimer levels (22), and the effect of rivaroxaban on D-dimer levels in AF did not differ from VKA (23, 24). However, differences in pathomechanism and epidemiology between AF and venous thrombosis might explain differences in the effect of DOACs and VKA on D-dimer levels in both disease entities.

Potential influencing factors of D-Dimer levels are age and sex, as D-dimer levels are correlated with increasing age (25–27) and are higher in women (28). As there was no difference in age and sex between patients on DOACs and VKA users in our study population, an interfering effect of age and sex on observed D-dimer levels is unlikely. In a comparison of the different types of venous thrombosis in the study population, the rate of isolated PE was higher in patients on apixaban than in patients on VKA, which might at least partially explain the difference in D-dimer levels between these cohorts. In the study of Legnani et al. the rate of patients with positive D-dimer during anticoagulant treatment was higher in patients on DOACs with DVT with or without PE, but not in DOAC users with isolated PE, in comparison to VKA users (17). In contrast to our study, only patients with a first thrombotic event in the absence of major risk factors were included in this previous study and different types of thrombosis were not separately assessed according to the different types of DOACs (17).

Due to the retrospective design of our study, DOACs or VKA were prescribed at the discretion of the treating physician, resulting in a real-world study population with an uneven distribution of the different anticoagulant drugs. Especially the cohorts of patients who received dabigatran and edoxaban were disproportionally small, precluding final conclusions. Factors not known to us might have influenced the selection of anticoagulant drugs by the treating physician, including hepatic or renal function, treatment adherence, or bleeding complications under preceding anticoagulant treatment. We refrained from analyzing various patient characteristics of interest, including comorbidities, clinical laboratory parameters, or adverse events, as these data were available only sporadically, owing to the retrospective nature of the study. Not all included DOACs were available on the market during the whole observation period, not all were indicated for immediate treatment of thrombosis, and not all dosages were indicated in the first months of anticoagulant therapy. To control for a potential selection bias due to these constraints, patients with a thrombosis within one month preceding D-dimer measurement, and patients receiving higher initial doses of apixaban and rivaroxaban were excluded. Furthermore, D-dimer levels were compared after stratification of the study population according to the time since the last thrombotic event. Further limitations of our study are that it is a single center study and D-dimer levels were measured using only one assay. Finally, D-dimer levels were not measured repeatedly within individuals, thus precluding a statement on the constancy of abnormal D-dimer levels in patients on DOACs.

The findings of increased D-dimer levels in patients on DOACs and of an increased rate of D-dimer levels might be explained by different modes of action of DOACs and VKA. Previously it has been shown that the direct thrombin inhibitor dabigatran increases, partially mediated by thrombin-activatable fibrinolysis inhibitor, the lysability of fibrin clots (29). Rivaroxaban, apixaban, and also the VKA warfarin have been shown to facilitate fibrin clot lysis (30, 31). Interestingly, it has been reported that the effect of DOACs on the fibrin clot properties can be reversed only partially by activated coagulation factor concentrate, in contrast to the effect of warfarin, which is completely neutralized (31). One might hypothesize, that differential effects of DOACs and VKA on fibrinolysis could explain the observed differences in plasma levels of D-dimer in our study population. Another potential explanation for the increased D-dimer levels in patients on DOACs could be a lower anticoagulation quality in comparison to patients on VKA. An adequate anticoagulation intensity was assured by INR testing in our reference cohort of DOAC users, but not in the cohorts treated with DOACs, as is also standard in routine clinical practice. Therefore, the inclusion of patients with poor anticoagulation quality, associated with higher D-dimer levels, into the DOAC cohorts might have led to an overall increase in D-dimer levels in comparison to the VKA cohort.

The results obtained in this study do not allow a conclusion on the risk of VTE recurrence in patients on DOACs and VKA. The usefulness of clinical decision algorithms that include D-dimer measurement in patients on DOACs for VTE risk stratification has been demonstrated previously (14). Our findings underline the importance to validate other algorithms, that include D-dimer measurements during anticoagulant treatment before their application in patients treated with DOACs.

## Data Availability Statement

The datasets presented in this article are not readily available because no concrete agreements on data sharing have been made yet. Before any data is shared, a data sharing plan will be drawn up, in consultation with the data officer and the ethics committee, that conforms to relevant laws and regulations concerning personal data. Requests to access the datasets should be directed to HR, heiko.ruehl@ukbonn.de.

## Ethics Statement

The studies involving human participants were reviewed and approved by the Institutional Review Board and Ethics Committee of the Medical Faculty of the University of Bonn. The patients/participants provided their written informed consent to participate in this study.

## Author Contributions

SR and HR contributed to the design of the manuscript and were the main authors. SR, ET, and HR collected clinical data. HR performed statistical analysis. ET, JM, NH, JO, and BP critically reviewed the manuscript. JM, NH, and BP contributed to the concept and design of the manuscript and revised the intellectual content. All authors contributed to the article and approved the submitted version.

## Conflict of Interest

The authors declare that the research was conducted in the absence of any commercial or financial relationships that could be construed as a potential conflict of interest.

## Publisher's Note

All claims expressed in this article are solely those of the authors and do not necessarily represent those of their affiliated organizations, or those of the publisher, the editors and the reviewers. Any product that may be evaluated in this article, or claim that may be made by its manufacturer, is not guaranteed or endorsed by the publisher.
